# Preparing entangled states between two NV centers via the damping of nanomechanical resonators

**DOI:** 10.1038/s41598-017-14245-8

**Published:** 2017-10-26

**Authors:** Xiao-Xiao Li, Peng-Bo Li, Sheng-Li Ma, Fu-Li Li

**Affiliations:** Shaanxi Province Key Laboratory of Quantum Information and Quantum Optoelectronic Devices, Department of Applied Physics, Xi’an Jiaotong University, Xi’an, 710049 China

## Abstract

We propose an efficient scheme for preparing entangled states between two separated nitrogen-vacancy (NV) centers in a spin-mechanical system via a dissipative quantum dynamical process. The proposal actively exploits the nanomechanical resonator (NAMR) damping to drive the NV centers to the target state through a quantum reservoir engineering approach. The distinct features of the present work are that we turn the detrimental source of noise into a resource and only need high-frequency low-Q mechanical resonators, which make our scheme more simple and feasible in experimental implementation. This protocol may have interesting applications in quantum information processing with spin-mechanical systems.

## Introduction

Hybrid quantum systems involving mechanical structures have attracted great interests in recent years^1–14^. Traditionally, mechanical systems have served as sensitive force detectors because of their sensitivity to electric, magnetic and optical fields. With the advance of nanofabrication techniques, mechanical motion has reached the quantum regime, thereby providing an ideal platform to test quantum theory with macroscopic objects. Furthermore, since the mechanical structure, often fabricated with a high quality factor Q (in the range of ~10^5^)^15^, can couple to a wide range of quantum systems, hybrid mechanical devices are now widely used in quantum information science. Thus far, experimental and theoretical progress has realized the coherent coupling between mechanical resonators and other quantum systems, such as superconducting circuits^16–20^, ultracold atoms^21–23^, quantum dots^24,25^, and solid-state defects^26–47^. This unconventional coupling offers a promising route for quantum-information processing with hybrid nanomechanical systems.

One of the most prominent examples of hybrid mechanical systems is the NV-nanomechanical system, where the coupling often arises from the relative motion of the NV center and a strongly magnetized tip. In such setups, either the NV center or the tip is affixed to a vibrating nanomechanical resonator^29–41^. The benefits of such hybrid quantum systems are quite diverse. For one thing, NV centers in diamond possess excellent coherence properties even at room temperature, and their electronic ground states can be tuned by external magnetic fields via Zeeman effect^48–56^. For another, several experiments have shown that the NV-mechanical coupling strength can reach 100 kHz, which far exceeds the decoherence rates of NV spins. Moreover, the interaction between an NV spin and a mechanical resonator can be described by the well-known Jaynes-Cummings (JC) Hamiltonian, in analogy with cavity quantum electrodynamics (cavity QED). In this case, mechanical resonators can be used as a quantum data bus to realize quantum states transfer between different quantum systems.

As for the application of NV-nanomechanical systems, generating entangled states between distant NV centers is of particular interest and experimental challenge. Several theoretical works have been proposed to prepare entangled states of NV centers by using a mechanical resonator as a data bus. L. G. Zhou *et al*.^38^ showed that the maximal entangled state between the NV centers can be probabilistically generated by detecting the frequency shift of the NAMR. Besides, L. Chotorlishvili *et al*.^41^ also proposed to steer two NV centers to the entangled state via control over the deflection of the cantilever. In essence, these schemes are based on the unitary dynamical evolution of the hybrid quantum system. However, the entanglement generated in these protocols will inevitably be affected by the mechanical damping and ambient thermal noises. The traditional method for beating such detrimental decoherence often needs the strong spin-phonon interaction to exceed the decay of the phonons. Then, the mechanical resonator must have an ultrahigh quality factor Q and often needs extra ground state cooling, but these requirements are too demanding for current nanofabrication techniques.

In this work, we propose a new scheme for generating entangled states of NV centers in an NV-nanomehanical system. In our setup, two NV centers are embedded separately in a nanomechanical resonator, and equally coupled to the mechanical motion through magnetic coupling. We apply a strong static magnetic field to the NV centers, enabling the NV spin transition |0〉 ↔ |−1〉 to be resonant with the mechanical mode. In addition, microwave driving fields are applied to each NV center in order to break the symmetry of the system Hamiltonian. As a result, the system will eventually evolve into a singlet-like entangled steady state^57^. Here, the damping of the mechanical resonator plays a positive role and helps drive the system to the target state, without the need of ultrahigh-Q nanomechanical resonators^58^. Similarly, the counterintuitive effects of loss have also been studied in optical systems, which shown that dissipation can be converted to gain near an exceptional point (EP)^59,60^. The distinct features of the present work are that we turn the detrimental source of noise into a resource and only need high-frequency low-Q mechanical resonators, which make our scheme more simple and feasible in experimental implementations. This work may have interesting applications in quantum information processing with spin-mechanical systems.

## Results

### The setup

**Figure 1:**
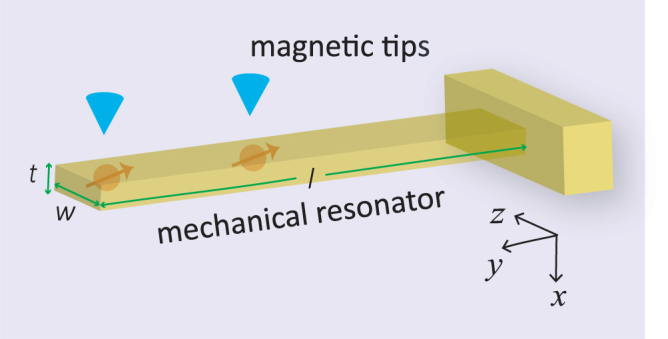
Schematics of the hybrid device studied in this work. Two NV centers are implanted separately in a nanomechanical resonator with dimensions (*l*, *w*, *t*). Magnetic tips are near the NV centers, which are used to generate strong magnetic gradients. The setup is immersed in a static magnetic field ${\vec{B}}_{z}$. Note that the crystalline axis of the NV center is along the z axis.

As shown in Fig. 1, two NV centers are implanted separately in a nanomechanical resonator, with the magnetic tips positioned at a distance *h* ~ 25 nm away from them. The setup is immersed in a static magnetic field in the positive direction of the *z* axis. The motion of the mechanical resonator along the *x* axis can be quantized, which is described by the Hamiltonian ${\hat{H}}_{r}=\hslash {\omega }_{r}{\hat{a}}^{\dagger }\hat{a}$, with $\hat{a}$ and ${\hat{a}}^{\dagger }$ the annihilation and creation operators for the vibrational mode.

As the mechanical resonator vibrates, the magnetic field felt by the NV centers will depend on their motion, which has the form $|{\vec{B}}_{tip}|\simeq {G}_{m}\hat{x}$, with *G*_*m*_ the magnetic field gradient, $\hat{x}={a}_{0}(\hat{a}+{\hat{a}}^{\dagger })$ the position operator, and *a*_0_ the mechanical zero-point amplitude^61^. For a cantilever with the mass density *ρ* and Young’s modulus *E*, the resonant frequency of the fundamental mode is ${\omega }_{r}=3.516\tfrac{t}{{l}^{2}}{(\tfrac{E}{12\rho })}^{\mathrm{1/2}}$ and the motional mass is *m*_*r*_ = *ρlwt*/4, where *l*, *w*, *t* refer to the length, width and thickness of the resonator, respectively^62^. In this paper, we consider a nanomechanical resonator with dimensions (*l*, *w*, *t*) = (200, 50, 50) nm, a magnetic field gradient *G*_*m*_ = 10^7^ T/m, and then the spin-phonon coupling strength can reach 2*π* × 30 kHz^63^. Apparently, this coupling strength considerably exceeds the effective decoherence rate of single NV spins.

**Figure 2:**
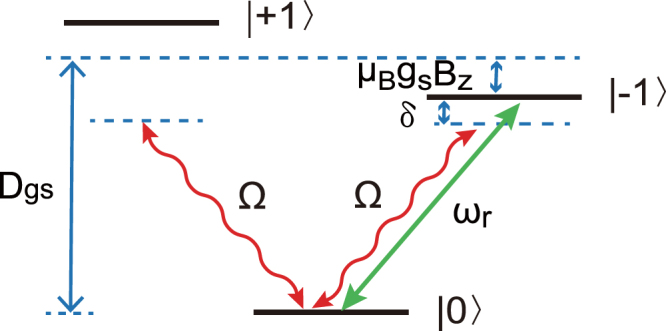
Simplified energy levels of a single NV center in the electronic ground state. Here, Ω denotes the Rabi frequencies between |0〉 and the excited states |±1〉, *μ*_*B*_ *g*_*s*_ *B*_*z*_ is the Zeeman shift under the static magnetic field, and *δ* denotes the detuning between the microwave driving field and the transition |0〉 ↔ |−1〉.

An NV center consists of a substitutional nitrogen atom with an adjacent vacancy in the diamond lattice, and the electronic ground states of a single NV center are spin triplet states denoted as |*m*_*s*_ = 0, ±1〉. The zero-field splitting *D*_*gs*_ between spin states with different values of |*m*_*s*_| is 2*π* × 2.87 GHz^64^. In our setup, the external magnetic fields applied to each NV center consist of three parts. One is static magnetic field ${\vec{B}}_{z}$, whose role is to cause Zeeman splitting of the states |*m*_*s*_ = ±1〉. And then the microwave fields are polarized in the *x* direction, ${\vec{B}}_{dr}={B}_{0}\,\cos \,{\omega }_{0}t{\vec{e}}_{x}$, which are applied to drive Rabi oscillations between |*m*_*s*_ = 0〉 and the excited state |*m*_*s*_ = ±1〉, as shown in Fig. 2. Besides, the gradient magnetic field ${\vec{B}}_{tip}={B}_{tip}{\vec{e}}_{x}$. Thus, the external magnetic fields can be written as ${\vec{B}}_{NV}={\vec{B}}_{z}+{\vec{B}}_{dr}+{\vec{B}}_{tip}$. The Hamiltonian of a single NV spin interacting with the total magnetic fields has the form

1 $${\hat{H}}_{NV}=\hslash {D}_{gs}{\hat{S}}_{z}^{2}+{\mu }_{B}{g}_{s}{\vec{B}}_{NV}\cdot \vec{S}\mathrm{.}$$

In the basis defined by the eigenstates of ${\hat{S}}_{z}$, i.e., {|*m*_*s*_〉, *m*_*s*_ = 0, ±1}, with ${\hat{S}}_{z}|{m}_{s}\rangle ={m}_{s}|{m}_{s}\rangle$, we get

2 $$\begin{array}{rcl}{\hat{H}}_{NV} & = & \hslash {D}_{gs}{m}_{s}^{2}|{m}_{s}\rangle \langle {m}_{s}|+{\mu }_{B}{g}_{s}{B}_{z}{m}_{s}|{m}_{s}\rangle \langle {m}_{s}|\\  &  & +\sum _{{m}_{s},{m}_{s}^{^{\prime} }}\,{\mu }_{B}{g}_{s}{B}_{0}\,\cos \,{\omega }_{0}t|{m}_{s}^{^{\prime} }\rangle \langle {m}_{s}^{^{\prime} }|{\hat{S}}_{x}|{m}_{s}\rangle \langle {m}_{s}|\\  &  & +\sum _{{m}_{s},{m}_{s}^{^{\prime} }}\,{\mu }_{B}{g}_{s}{G}_{m}{a}_{0}(\hat{a}+{\hat{a}}^{\dagger })|{m}_{s}^{^{\prime} }\rangle \langle {m}_{s}^{^{\prime} }|{\hat{S}}_{x}|{m}_{s}\rangle \langle {m}_{s}|.\end{array}$$

In this paper, we assume that two NV centers are equally coupled to the cantilever (*λ* = *λ*_*i*_), which could be realized by skillfully adjusting the magnetic field gradient of the tips and the distances between the tips and the corresponding NV centers. The direct spin-spin interaction can be neglected since it is excessively weak compared with the spin-phonon interaction^65^. Besides, we consider the NV centers can be individually addressed. Note that, for spin-1 system, the spin operator component ${\hat{S}}_{x}=\frac{\hslash }{\sqrt{2}}(|0\rangle \langle +1|+|0\rangle \langle -1|+H.c.)$. The Hamiltonian of the system then takes the form

3 $$\begin{array}{rcl}{H}_{S} & = & \hslash {\omega }_{r}{\hat{a}}^{\dagger }\hat{a}+\sum _{i=1,2}\,[\hslash {{\rm{\Delta }}}_{-}{|-1\rangle }_{i}{\langle -1|+\hslash {{\rm{\Delta }}}_{+}|+1\rangle }_{i}\langle +1|\\  &  & +\hslash {{\rm{\Omega }}}_{i}({|0\rangle }_{i}{\langle +1|+|0\rangle }_{i}\langle -1|+H\mathrm{.}c\mathrm{.})\,({e}^{i{\omega }_{i0}t}+{e}^{-i{\omega }_{i0}t})\\  &  & +\hslash \lambda ({|0\rangle }_{i}{\langle +1|+|0\rangle }_{i}\langle \,-1|+H\mathrm{.}c\mathrm{.})\,(\hat{a}+{\hat{a}}^{\dagger })],\end{array}$$

where *ħ*Δ_± _= *ħD*_*gs*_ ± *μ*_*B*_ *g*_*s*_ *B*_*z*_, $\hslash {{\rm{\Omega }}}_{i}=\tfrac{\sqrt{2}}{4}{\mu }_{B}{g}_{s}{B}_{i0}$, $\hslash \lambda =\tfrac{\sqrt{2}}{2}{\mu }_{B}{g}_{s}{G}_{m}{a}_{0}$, *λ* is the coupling strength between the NV centers and the NAMR, and *ω*_*i*0_ are the frequencies of microwave driving fields.

If we consider the resonance condition, *ω*_*r*_ ≈ Δ_−_, then the transition between the states |0〉 and |+1〉 is far off-resonance for the mechanical mode and therefore can be removed from Eq. (3). Besides, we assume *ω*_*i*0_ ≈ Δ_−_, i.e, the microwave fields contain only one frequency, which near-resonant to the transition |0〉 ↔ |−1〉. If the free Hamiltonian is chosen as ${H}_{0}=\hslash {\omega }_{r}{\hat{a}}^{\dagger }\hat{a}+{\sum }_{i=1,2}\,(\hslash {\omega }_{i0}{|-1\rangle }_{i}{\langle -1|+\hslash {{\rm{\Delta }}}_{+}|+1\rangle }_{i}\langle +1|)$, under the rotating-wave approximation, we can obtain the interaction Hamiltonian

4 $$\begin{array}{rcl}{H}_{I} & = & \hslash {\delta }_{1}{|-1\rangle }_{1}{\langle -1|+\hslash {\delta }_{2}|-1\rangle }_{2}\langle -1|\\  &  & +\hslash {\rm{\Omega }}({|0\rangle }_{1}{\langle -1|+|0\rangle }_{2}\langle -1|+H\mathrm{.}c\mathrm{.})\\  &  & +\hslash \lambda ({\hat{a}}^{\dagger }{|0\rangle }_{1}{\langle -1|+{\hat{a}}^{\dagger }|0\rangle }_{2}\langle -1|+H\mathrm{.}c\mathrm{.}),\end{array}$$

with *δ*_*i*_ = Δ_−_ − *ω*_*i*0_, and here we have assumed Ω_*i*_ = Ω. From the Hamiltonian (4), we can find that the spin-phonon interaction is analogous to the Jaynes-Cummins model of two-level atoms coupled to a single cavity mode, with resonant phonons playing the role of cavity photons. Therefore, similar to previous works in cavity QED^66^, our setup can achieve the preparation of the entangled states between two NV centers through a dissipative quantum dynamical process.

### The dissipative quantum dynamical process

In this section, we show how to drive the NV centers to an entangled stationary state through a quantum reservoir engineering approach. Taking into account the coupling of the NAMR with the environment in the Markovian approximation, we can write the master equation for the density operator $\hat{\rho }$ of the system in the form

5 $$\frac{d\hat{\rho }}{dt}=-\frac{i}{\hslash }[{H}_{I},\hat{\rho }]+\sum _{i=\mathrm{1,2}}\,{\gamma }_{s}D[{\sigma }_{z}^{i}]\hat{\rho }+{n}_{th}{\gamma }_{m}D[{\hat{a}}^{\dagger }]\hat{\rho }+({n}_{th}+\mathrm{1)}{\gamma }_{m}D[\hat{a}]\hat{\rho },$$

with ${\hat{\sigma }}_{z}^{i}={|-1\rangle }_{i}{\langle -1|-|0\rangle }_{i}\langle 0|$, *γ*_*s*_ the spin dephasing rate of the NV centers, *γ*_*m*_ = *ω*_*r*_/*Q* the intrinsic damping rate of the mechanical resonator, and $D[\hat{o}]\hat{\rho }=\hat{o}\hat{\rho }{\hat{o}}^{\dagger }-\tfrac{1}{2}\hat{\rho }{\hat{o}}^{\dagger }\hat{o}-\tfrac{1}{2}{\hat{o}}^{\dagger }\hat{o}\hat{\rho }$ for a given operator $\hat{o}$. Here, we focus on the regime ${\gamma }_{m}\gg {\gamma }_{s}$, and ignore the dissipation of the NV centers in the following. Besides, in the regime of large mechanical frequency (in the gigahertz range) and at cryogenic temperature, the thermal phonon number is nearly zero, i.e., ${n}_{th}={({e}^{\hslash {\omega }_{r}/{k}_{B}T}-1)}^{-1}\simeq 0$, which corresponds to coupling with the vacuum bath for the NAMR. Then Eq. (5) reduces to

6 $$\frac{d\hat{\rho }}{dt}=-\frac{i}{\hslash }[{H}_{I},\hat{\rho }]+{\gamma }_{m}D[\hat{a}]\hat{\rho }\mathrm{.}$$

We now introduce the phonon number representation for the density operator $\hat{\rho }$ with respect to the vibrational mode $\hat{a}$, i.e., $\hat{\rho }={\sum }_{m,n=0}^{\infty }\,{\rho }_{mn}|m\rangle \langle n|$, where *ρ*_*mn*_ are the field-matrix elements in the basis of the phonon number states. Under the condition of strong mechanical damping, we can neglect populations of the highly excited modes. Thus, we consider only the matrix elements *ρ*_*mn*_ inside the subspace {|0〉, |1〉} of the phonon numbers^67^. Under this approximation, the master equation (6) leads to the following set of coupled equations of motion for the density-matrix elements (let *ħ* = 1)

7 $$\begin{array}{rcl}{\dot{\rho }}_{00} & = & \hat{F}{\rho }_{00}+i\lambda {\rho }_{01}{|0\rangle }_{1}{\langle -1|+i\lambda {\rho }_{01}|0\rangle }_{2}{\langle -1|-i\lambda |-1\rangle }_{1}{\langle 0|{\rho }_{10}-i\lambda |-1\rangle }_{2}\langle 0|{\rho }_{10}+{\gamma }_{m}{\rho }_{11},\\ {\dot{\rho }}_{11} & = & \hat{F}{\rho }_{11}+i\lambda {\rho }_{10}{|-1\rangle }_{1}{\langle 0|+i\lambda {\rho }_{10}|-1\rangle }_{2}{\langle 0|-i\lambda |0\rangle }_{1}{\langle -1|{\rho }_{01}-i\lambda |0\rangle }_{2}\langle -1|{\rho }_{01}-{\gamma }_{m}{\rho }_{11},\\ {\dot{\rho }}_{01} & = & \hat{F}{\rho }_{01}+i\lambda {\rho }_{00}{|-1\rangle }_{1}{\langle 0|+i\lambda {\rho }_{00}|-1\rangle }_{2}{\langle 0|-i\lambda |-1\rangle }_{1}{\langle 0|{\rho }_{11}-i\lambda |-1\rangle }_{2}\langle 0|{\rho }_{11}-\tfrac{{\gamma }_{m}}{2}{\rho }_{01},\\ {\dot{\rho }}_{10} & = & \hat{F}{\rho }_{10}+i\lambda {\rho }_{11}{|0\rangle }_{1}{\langle -1|+i\lambda {\rho }_{11}|0\rangle }_{2}{\langle -1|-i\lambda |0\rangle }_{1}{\langle -1|{\rho }_{00}-i\lambda |0\rangle }_{2}\langle -1|{\rho }_{00}-\tfrac{{\gamma }_{m}}{2}{\rho }_{10},\end{array}$$

where

8 $$\begin{array}{rcl}\hat{F}{\rho }_{ij} & = & -i[{\delta }_{1}{|-1\rangle }_{1}{\langle -1|+{\delta }_{2}|-1\rangle }_{2}\langle -1|+{\rm{\Omega }}({|-1\rangle }_{1}\langle 0|\\  &  & +{|0\rangle }_{1}{\langle -1|+|-1\rangle }_{2}{\langle 0|+|0\rangle }_{2}\langle -1|),{\rho }_{ij}]\mathrm{.}\end{array}$$

Note that the field-matrix elements *ρ*_*mn*_ are still operators with respect to the NV centers. Then the reduced density operator for the NV centers can be approximated as ${\hat{\rho }}_{NV}=T{r}_{M}(\hat{\rho })\simeq {\rho }_{00}+{\rho }_{11}$. Since the vibrational mode is strongly damped, most of the phonons are in the ground state |0〉. Then we can assume that the coherence *ρ*_01_ and *ρ*_10_ change slowly in time, so that we can take ${\dot{\rho }}_{01}=0$, and ${\dot{\rho }}_{10}=0$. In this case, the elements *ρ*_11_ can be neglected from Eq. (7), and then the master equation for the reduced density operator of the two NV centers takes the form

9 $$\frac{d{\hat{\rho }}_{NV}}{dt}=-i[{H}_{d},{\hat{\rho }}_{NV}]+L{\hat{\rho }}_{NV}{L}^{\dagger }-\frac{1}{2}({L}^{\dagger }L{\hat{\rho }}_{NV}+{\hat{\rho }}_{NV}{L}^{\dagger }L),$$

where

10 $${H}_{d}={\delta }_{1}{|-1\rangle }_{1}{\langle -1|+{\delta }_{2}|-1\rangle }_{2}\langle -1|+{\rm{\Omega }}({|-1\rangle }_{1}{\langle 0|+|-1\rangle }_{2}\langle 0|+H\mathrm{.}c\mathrm{.}),$$

11 $$L=\sqrt{\frac{4{\lambda }^{2}}{{\gamma }_{m}}}({|0\rangle }_{1}{\langle -1|+|0\rangle }_{2}\langle \,-1|)\mathrm{.}$$

We find that Eq. (9) is the standard form of the master equation. The first term describes the interaction of the NV centers and the microwave driving fields, while the last two terms represent an effective engineered reservoir for the NV centers.

Now we introduce collective states for the two NV centers, i.e., the ground state |*G*〉 = |0〉_1_ |0〉_2_, the upper state |*E*〉 = |−1〉_1_ |−1〉_2_, the symmetric state $|T\rangle =\frac{1}{\sqrt{2}}({|0\rangle }_{1}{|-1\rangle }_{2}+{|-1\rangle }_{1}{|0\rangle }_{2})$ and the antisymmetric state $|S\rangle =\frac{1}{\sqrt{2}}({|0\rangle }_{1}{|-1\rangle }_{2}-{|-1\rangle }_{1}{|0\rangle }_{2})$, of which |*T*〉 and |*S*〉 are maximally entangled states^67^. In order to make the evolution of the system more clear, we transform the Hamiltonian *H*_*d*_ and the Lindblad operator *L* into the basis of the collective states. Then we can obtain

12 $${H}_{d}=\sqrt{2}{\rm{\Omega }}|E\rangle \langle T|+\sqrt{2}{\rm{\Omega }}|T\rangle \langle G|-\,\delta |S\rangle \langle T|+H\mathrm{.}c\mathrm{.}$$

13 $$L=\sqrt{\frac{8{\lambda }^{2}}{{\gamma }_{m}}}(|G\rangle \langle T|+|T\rangle \langle E|)\mathrm{.}$$

**Figure 3:**
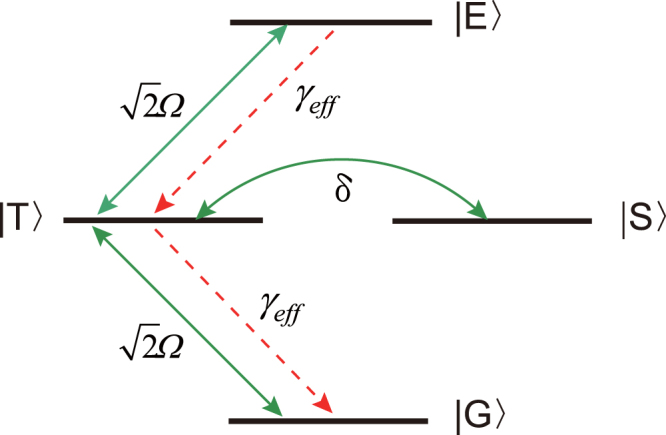
Effective process of the scheme. The microwave driving $\sqrt{2}{\rm{\Omega }}$ induces transitions of |*G*〉 ↔ |*T*〉 and |*T*〉 ↔ |*E*〉. The effective damping rate *γ*_*eff*_ causes transitions of |*G*〉 → |*T*〉 and |*T*〉 → |*B*〉. The state |*S*〉 is coherently coupled to |*T*〉 by the level shift *δ*.

Here, we have assumed *δ*_1_ = −*δ*_2_ = *δ*, ie., two microwave fields are red detuned and blue detuned by *δ* respectively. The effective processes described by the Hamiltonian *H*_*d*_ and the Lindblad operator *L* are shown in Fig. 3. According to the Hamiltonian *H*_*d*_, the microwave driving fields can induce transitions |*G*〉 ↔ |*T*〉, |*T*〉 ↔ |*E*〉 and |*T*〉 ↔ |*S*〉. On the other hand, the Lindblad operator *L* will drive |*G*〉 → |*T*〉 and |*T*〉 → |*E*〉 with an effective decay rate $8{\lambda }^{2}/{\gamma }_{m}$. In the following, by calculating the eigenstate with eigenvalue 0 of the Hamiltonian *H*_*d*_ and the Lindblad operator *L*, we can obtain a unique steady state of the system

14 $$|{\psi }_{S}\rangle ={\eta }^{-1}(\delta |G\rangle +\sqrt{2}{\rm{\Omega }}|S\rangle )$$

where $\eta =\sqrt{{\delta }^{2}+2{{\rm{\Omega }}}^{2}}$ is the normalized coefficient. As discussed above, two NV centers can be steered into a steady entangled state by utilizing the dissipation of the NAMR. In the following section, we can confirm the results by means of numerical simulations.

Note that the preparation of entangled states of two NV centers in the hybrid mechanical system can also be achieved by using the unitary dynamical evolution of the system^30,38^. These schemes work in the large detuning regime, $\delta =|{{\rm{\Delta }}}_{-}-{\omega }_{i0}|\gg \lambda$, and then the two NV centers can couple to each other via the exchange of virtual phonons. According to the calculation, this phonon-mediated spin-spin coupling strength can be given by *λ* ^2^/*δ*. For our proposal, the microwave fields and the transition |0〉 ↔ |−1〉 are near-resonance. The spin-spin entanglement results from the interaction between two NV centers and a common engineered reservoir.

### Numerical simulations

In order to verify the model and evaluate the performance of this protocol, we simulate the dynamics of the system by solving the full master equation (5) numerically^68^. In the calculation, we choose *δ*_1_ = −*δ*_2_ = 0.1*λ*, Ω = 0.1*λ* and the mechanical resonator damping rate *γ*_*m*_ = 20*λ*. These parameters are chosen in such a way that they are within the parameter range for which this scheme is valid and are accessible with current experimental setup. Note that the above analysis has neglected the dephasing effect of the NV centers, however, which should be considered under the realistic circumstance. In the following, we calculate the time evolution of the population, fidelity and concurrence of the system for different initial states.

**Figure 4:**
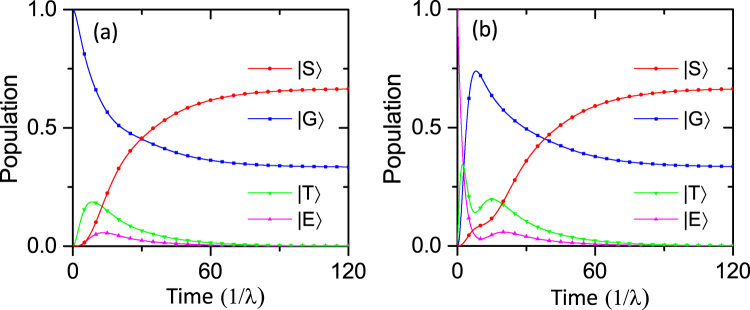
Time evolution of the population *P* of the collective states from two different initial states (**a**) |*G*〉, (**b**) |*E*〉. The relevant parameters are chosen as *δ*_1_ = −*δ*_2_ = 0.1*λ*, Ω = 0.1*λ*, *n*_*th*_ = 0.01, *γ*_*m*_ = 10*λ* and *γ*_*s*_ = 0.

Figure 4 shows the numerical results for the populations of the collective states |*G*〉, |*E*〉, |*T*〉 and |*S*〉 without spin dephasing. In this case, the evolution of the system follows Eq. (12), and the combined effect of the Hamiltonian *H*_*d*_ and Lindblad operator *L* drives the system to the unique stationary state |*ψ*_*S*_〉. From this figure we find that, when the system reaches the steady state, the probabilities for detecting the states |*G*〉 and |*S*〉 approximate to 0.33 and 0.67, respectively, and the probabilities for detecting the states |*E*〉 and |*T*〉 are almost 0. These numerical results are highly consistent with the previous analysis under the above choice of parameters. Moreover, we can also find that the probability of the steady state is independent of the initial states. Thus, the numerical simulations for populations prove our scheme valid.

**Figure 5:**
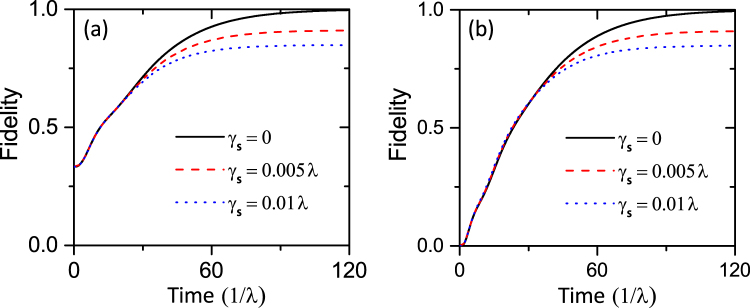
Time evolution of the fidelity *F* from two different initial states (**a**) |*G*〉, (**b**) |*E*〉. The dephasing rate *γ*_*s*_ are chosen as 0, 0.005*λ* and 0.01*λ*. Other parameters are the same as those in Fig. 4.

The fidelity with respect to the state |*ψ*_*S*_〉 is defined as *F* = 〈*ψ*_*S*_|*ρ*_*NV*_(*t* → ∞)|*ψ*_*S*_〉, where *ρ*_*NV*_ is the reduced density operator for the NV centers. In Fig. 5, we simulate the fidelity *F* as a function of time starting from two different initial states |*G*〉 and |*E*〉. In the absence of spin dephashing of the NV centers, it is shown that, the system evolves to the stationary states |*ψ*_*S*_〉 with a fidelity higher than 99.9% (black solid line). This simulation result indicates that the two NV centers are indeed driven into the target state. In addition, we also simulate time evolution of the fidelity *F* taking into consideration of spin dephasing. As shown in Fig. 5, when setting the dephashing rate *γ*_*s*_ = 0.005*λ*, it is seen that the fidelity *F* = 91%. Furthermore, as the dephashing rate increases to 0.01*λ*, which is close to the realistic experimental conditions, the fidelity of this scheme can still reach 85% (blue dash line). Therefore, our protocol can achieve high fidelity of the target state with feasible experimental parameters.

**Figure 6:**
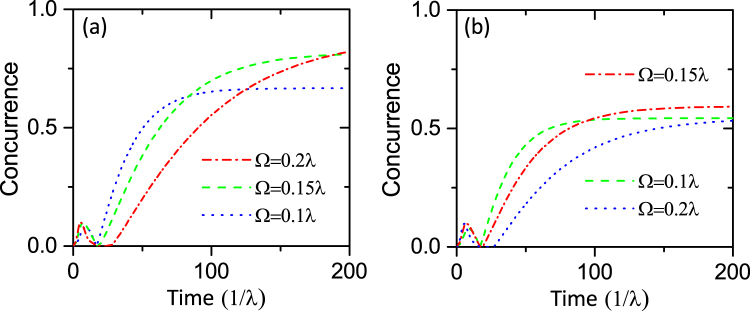
Time evolution of the concurrence *C* with different Ω. (**a**) *γ*_*s*_ = 0, (**b**) *γ*_*s*_ = 0.005*λ*. Other parameters are the same as those in Fig. 4.

According to ref.^69^, we can calculate the concurrence of the steady state as *C*_*S*_ = 2Ω^2^/*η* ^2^. Then the entanglement between the NV centers can be enhanced by improving the value of Ω. In order to assess the entanglement between the NV centers, we simulate the time evolution of the concurrence as shown in Fig. 6. We first consider the ideal case in which the spin dephasing is neglected. As illustrated in Fig. 6(a), the concurrence is close to 0.67 when we take Ω = 0.1*λ* (blue dot line), and the concurrence has a significant increase when Ω takes the larger values. At the same time, we can see that the time for reaching the stationary state has also increased. However, to implement this proposal with high concurrence, the time required for the system to reach the steady state should be shorter than the coherence time of single NV spins. Furthermore, when taking the spin dephasing into consideration, we notice that there is obvious decrease in the concurrence when Ω = 0.2*λ*. Thus, when improving the value of Ω, we should consider the impact of spin dephasing as well.

**Figure 7:**
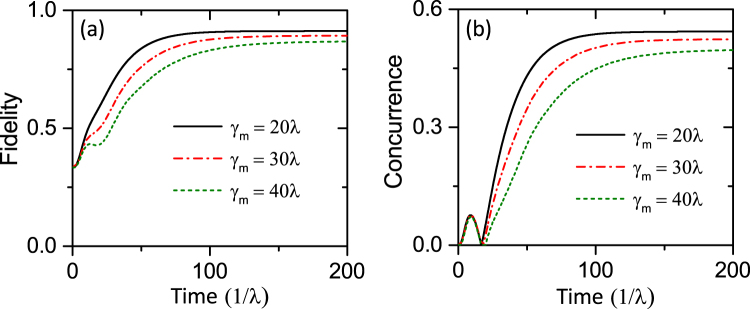
Time evolution of the fidelity *F* and concurrence *C* with different *γ*_*m*_. The spin dephasing rate *γ*_*s*_ = 0.005*λ*, and other parameters are the same as those in Fig. 4.

In addition, we simulate the impact of different mechanical damping rates on the fidelity and concurrence. In this case, the spin dephasing and other parameters remain unchanged, while the damping rates of the resonator are taken as 20*λ*, 30*λ*, 40*λ*. As illustrated in Fig. 7, with the increase of the mechanical damping rate, the fidelity and concurrence of the steady state are obviously reduced. Moreover, for the larger oscillator dissipation, it takes a longer time for the system to reach the steady state. Actually, this simulation results are exactly in agreement with the above theoretical derivation. From Eq. (13) the effective decay rate is inversely proportional to the mechanical damping rate *γ*_*m*_, i.e., as the oscillator dissipation increases, the time for the system to reach the final steady state will be longer. However, to make sure this scheme is valid, the time required for the system to reach the stationary state should be shorter than the coherence time of single NV spins. From Fig. 7 we can find that when the mechanical damping rates satisfies *γ*_*s*_ < *λ* ^2^/*γ*_*m*_, the time for the system to reach the steady state is acceptable. Therefore, in order to make sure our scheme is valid, there is a limit for the mechanical damping rate, i.e., *γ*_*m*_ < *λ* ^2^/*γ*_*s*_.

## Experimental Consideration

Finally, we proceed to examine the feasibility of our scheme in a realistic experiment. In our scheme, the static magnetic field *B*_*z*_ is about 0.05 T, which is easy to achieve in the present-day experiment^70^. As discussed previously, we consider a nanomechanical resonator with dimensions (*l*, *w*, *t*) = (200, 50, 50) nm, the mass ${m}_{r}\simeq 3\times {10}^{-19}\,{\rm{kg}}$, and ${a}_{0}=\sqrt{\hslash /2{m}_{r}{\omega }_{r}}\simeq 1.4\times {10}^{-13}\,{\rm{m}}$. Then the coupling strength between the NV centers and the NAMR is about 2*π* × 30 kHz. Obviously, this coupling strength considerably exceeds the single NV center decoherence rate with a coherence time (*T*_2_ ~ 1 ms). The fundamental mode of the mechanical resonator is ${\omega }_{r}/2\pi \simeq 1.5\,{\rm{GHz}}$, from which we obtain the damping rate ${\gamma }_{m}/2\pi \simeq 600\,{\rm{kHZ}}$ (*Q* = 2.5 × 10^3^). In this case, the damping of the NAMR can be utilized to drive the system to the target state. At the temperature $T\simeq 10\,{\rm{mK}}$, the equilibrium phonon occupation number is less than 0.01, and it can be neglected from the master equation (5). According to the numerical results, when we take *δ* = 0.1*λ*, Ω = 0.1*λ*, the time for the system to reach the stationary state |*ψ*_*S*_〉 is about 120/*λ* ~ 0.6 ms, which is shorter than the coherence time *T*_2_. Moreover, if we choose an appropriate value for Ω, the entanglement between the NV centers can be enhanced. Therefore, with current technologies in the field of spin-mechanics, this proposal can be implemented in experiment.

## Conclusion

We have proposed an efficient scheme for generating entangled states between two spatially separated NV centers which are coupled by a nanomechanical resonator. With the vibration of the mechanical resonator, a motion-dependent magnetic field is applied to the NV center spins, which induces a strong-coupling between the mechanical resonator and the NV centers. In our scheme, the preparation of the entangled state is based on a dissipative quantum dynamical process, which converts the intrinsic damping of the mechanical resonator into a resource. Compared to previous works utilizing unitary dynamical process, our scheme does not need the specific preparation of the initial state and designed special dynamical process of the system. More importantly, our setup only needs high-frequency low-Q mechanical resonators, which has significant advantages in actual experiments. With the advanced technology for nanomechanical resonators, this protocol can offer a realistic platform for implementing quantum information with spin-mechanical systems.
